# Filarial Lymphedema Is Characterized by Antigen-Specific Th1 and Th17 Proinflammatory Responses and a Lack of Regulatory T Cells

**DOI:** 10.1371/journal.pntd.0000420

**Published:** 2009-04-21

**Authors:** Subash Babu, Sajid Q. Bhat, N. Pavan Kumar, Angelo B. Lipira, Sanath Kumar, C. Karthik, V. Kumaraswami, Thomas B. Nutman

**Affiliations:** 1 National Institutes of Health–International Center for Excellence in Research, Chennai, India; 2 SAIC–Frederick, Inc., NCI-Frederick, Frederick, Maryland, United States of America; 3 Tuberculosis Research Center, Chennai, India; 4 Laboratory of Parasitic Diseases, National Institute of Allergy and Infectious Diseases, National Institutes of Health, Bethesda, Maryland, United States of America; Federal University of Minas Gerais, Brazil

## Abstract

**Background:**

Lymphatic filariasis can be associated with development of serious pathology in the form of lymphedema, hydrocele, and elephantiasis in a subset of infected patients.

**Methods and Findings:**

To elucidate the role of CD4^+^ T cell subsets in the development of lymphatic pathology, we examined specific sets of cytokines in individuals with filarial lymphedema in response to parasite antigen (BmA) and compared them with responses from asymptomatic infected individuals. We also examined expression patterns of Toll-like receptors (TLR1–10) and Nod-like receptors (Nod1, Nod2, and NALP3) in response to BmA. BmA induced significantly higher production of Th1-type cytokines—IFN-γ and TNF-α—in patients with lymphedema compared with asymptomatic individuals. Notably, expression of the Th17 family of cytokines—IL-17A, IL-17F, IL-21, and IL-23—was also significantly upregulated by BmA stimulation in lymphedema patients. In contrast, expression of Foxp3, GITR, TGFβ, and CTLA-4, known to be expressed by regulatory T cells, was significantly impaired in patients with lymphedema. BmA also induced significantly higher expression of TLR2, 4, 7, and 9 as well Nod1 and 2 mRNA in patients with lymphedema compared with asymptomatic controls.

**Conclusion:**

Our findings implicate increased Th1/Th17 responses and decreased regulatory T cells as well as regulation of Toll- and Nod-like receptors in pathogenesis of filarial lymphedema.

## Author Summary

Lymphatic filariasis afflicts over 120 million people worldwide. While the infection is mostly clinically asymptomatic, approximately 40 million people suffer from overt, morbid clinical pathology, characterized by swelling of the scrotal area and lower limbs (hydrocele and lymphedema). Host immunologic factors that determine susceptibility of this subset of individuals to clinically apparent disease is unknown. We addressed this issue by comparing the immunologic response of helper T (Th) cell subsets in lymphedema patients and clinically asymptomatic individuals. Four important subsets of Th cells (CD4^+^ T cells) are: Th1 cells, characterized by production of cytokines IL-2, IFN-γ, and TNF-α; Th2 cells, characterized by production of cytokines IL-4 and IL-13; Th17 cells, characterized by production of cytokines IL-17A, IL-17F, IL-21, and IL-22; and regulatory T cells (Tregs), characterized by the expression of transcription factor Foxp3, as well as other molecules such as GITR, TGFβ, and CTLA-4. We determined that Th1 and Th17 responses are significantly enhanced and Treg responses significantly impaired in lymphedema patients. This is accompanied by increased expression of pattern recognition receptors of the Toll and NOD family. Thus, approaches toward downregulating Th1 and Th17 responses and enhancing Treg responses would be beneficial in protecting against development of lymphatic disease.

## Introduction

Although two-thirds of the 120 million people infected with *Wuchereria bancrofti*, the major causative agent of human lymphatic filariasis, have subclinical infections, ∼40 million have lymphedema and/or other pathologic manifestations including hydroceles (and other forms of urogenital disease), episodic adenolymphangitis, tropical pulmonary eosinophilia, lymphedema, and (in its most severe form) elephantiasis [1]. Adult *W. bancrofti* worms reside in the lymphatics and lymph nodes and induce changes that result in dilatation of lymphatics and thickening of the lymphatic vessel walls. Progressive lymphatic damage and pathology results from the summation of the effect of tissue alterations induced by both living and nonliving adult parasites, the host inflammatory response to the parasites and their secreted antigens, the host inflammatory response to the endosymbiont Wolbachia, and those seen as a consequence of secondary bacterial or fungal infections [2]–[4].

Lymphatic damage in animal models of filarial infection has been shown to be dependent on T cells. Thus, nude mice (lacking T cells) and SCID mice (lacking T and B cells) exhibit only reversible, moderate pathology that becomes irreversible and severe upon T cell reconstitution [5],[6]. Moreover, T cells have also been shown to play a role in human lymphatic disease, with the presence of abnormal T cell infiltrates exhibiting a biased TCR repertoire at the site of inflammation [7],[8]. T cells in lymphedema patients are also characterized by abnormal chemokine receptor expression, with unusually high expression of CCR9 as well as increased surface expression of HLA-DR and VCAM-1 [9]–[11]. In addition to T cells, pattern recognition receptors of the Toll-like receptor (TLR) family—most notably TLR2 and TLR4—have been implicated in the development of filarial pathology [12]. In an animal model of a closely related filarial infection, onchocerciasis, the primary cause of ocular pathology has been suggested to be based on the interaction of the bacterial endosymbiont Wolbachia with host TLR4 [13].

Downregulation of TLR on antigen-presenting cells (APCs) and T cells has been shown to be a possible mechanism by which deleterious pathology in clinically asymptomatic filarial infections can be circumvented [14],[15]. Thus, inflammatory damage induced by filarial parasites appears to be multifactorial, with endogenous parasite products, Wolbachia, and host immunity all playing important roles. The role of regulatory T cells (Treg)—known to suppress inflammation in a variety of inflammatory and infectious diseases—in modulating pathology caused by human lymphatic filariasis is not known.

While the nature of immune responses to parasite antigens has been well characterized in patients with the asymptomatic (or subclinical) form of filarial infection, the type of T cell responses (Th1/Th2/Th17/natural Treg [nTreg]) that characterizes development of filarial pathology is less well studied. We therefore examined the expression patterns of Th1 (IL-2, IFN-γ, and TNF-α), Th2 (IL-4 and IL-13) and IL-10, Th17 (IL-17A, IL-17B, IL-21, IL-22, and IL-23), and nTreg (Foxp3, GITR, TGFβ, and CTLA-4) markers in filarial lymphedema patients and asymptomatic infected individuals following stimulation with parasite and control antigens. In addition, because of the putative causative role of TLRs in animal models of filarial pathology, we have examined the regulation of TLR expression as well as the expression of a closely related pattern recognition receptor family—Nod-like receptor (NLR).. We show that lymphatic disease in human filariasis is characterized by increased antigen-driven Th1 and Th17 cytokine induction, impaired Treg induction, and enhanced mRNA expression of both TLR and NLR family members.

## Methods

### Study population

We studied a group of 12 patients with filarial lymphedema (hereafter CP) and 10 asymptomatic infected (hereafter INF) patients in an area endemic for lymphatic filariasis in Tamil Nadu, South India (Table 1). CP patients were circulating filarial antigen negative by both the ICT filarial antigen test (Binax, Portland, ME) and the TropBio Og4C3 enzyme-linked immunosorbent assay (ELISA) (Trop Bio Pty. Ltd, Townsville, Queensland, Australia), indicating lack of current active infection. They had undergone treatment with repeated doses of diethylcarbamazine. The diagnosis of prior filarial infection was made by history and clinical examination as well as positive *Brugia malayi* antigen (BmA)-specific IgG4. BmA-specific IgG4 and IgG ELISA were performed exactly as described previously [16]. INF patients tested positive for active infection by both the ICT filarial antigen test and the TropBio Og4C3 ELISA. All individuals were examined as part of a clinical protocol approved by Institutional Review Boards of both the National Institutes of Allergy and Infectious Diseases and the Tuberculosis Research Center, and informed written consent was obtained from all participants.

**Table 1:** Characteristics of the study population.

	Filarial lymphedema (n = 12)	Infected (n = 10)
Median Age (Range)	49 (38–63)	32 (21–50)
Gender M / F	8 / 4	6 / 4
Treatment	Yes	No
Pathology	Grade 2 Lymphedema – 4	None
	Grade 3 Lymphedema –4	
	Grade 4 Lymphedema – 4	
*W. bancrofti* circulating antigen levels U/ml* (Median)	<32 (<32)	1307–32768 (18305)
BmA-specific IgG (µg/ml) (GM)	1.51–82.44 (11.74)	17.1–152.6 (54.2)
BmA-specific IgG4 (ng/ml) (GM)	441–2072 (810.4)	4293–21566 (9432.4)

*: The lower limit of the assay detection was 32 U/ml.

### Isolation of peripheral blood mononuclear cells (PBMCs)

Heparinized blood was collected and PBMCs isolated by Ficoll diatrizoate gradient centrifugation (LSM; ICN Biomedicals, Aurora, OH). Erythrocytes were lysed using ACK lysis buffer (Biosource International, Camarillo, CA). Cells were then washed and cultured in RPMI-1640 (BioWhittaker, Walkersville, MD) supplemented with 20 mM glutamine (BioWhittaker), 10% heat-inactivated FCS (Harlan Bioproducts for Science), and 50 µg/ml of gentamycin (Mediatech, Herndon, VA).

### Parasite and control antigen

Saline extracts of *B. malayi* adult worms (BmA) were used as the parasite antigen and mycobacterial PPD (Serum Statens Institute, Copenhagen, Denmark) was used as the control antigen. Final concentrations were 10 µg/ml for BmA and 10 µg/ml for PPD. Endotoxin level of final soluble BmA was <0.1 EU/ml using the QCL-1000 Chromogenic LAL test kit (BioWhittaker).

### In vitro culture

PBMCs were cultured with BmA or PPD in 24-well tissue culture plates (Corning, Corning, NY) at concentrations of 5×10^6^/well. After 24 hours, culture supernatants were collected and analyzed for cytokines.

### ELISA

The levels of cytokines in the culture supernatants were measured using Bioplex multiplex cytokine assay system (Biorad, Hercules, CA). The cytokines analyzed were IL-2, IFN-γ, TNF-α, IL-4, IL-6, IL-10, and IL-13.

### RNA preparation

RNA was isolated from PBMC following culture with BmA or PPD for 24 h. PBMCs were lysed using the reagents of a commercial kit (QIAshredder; Qiagen, Valencia, CA). Total RNA was extracted according to the manufacturer's protocol (RNeasy mini kit; Qiagen), and RNA was dissolved in 50 µl of RNase-free water.

### cDNA synthesis

RNA (1 µg) was used to generate cDNA using TaqMan reverse transcription reagents according to the manufacturer's protocol (Applied Biosystems, Fullerton, CA). Briefly, random hexamers were used to prime RNA samples for reverse transcription using MultiScribe reverse transcriptase.

### Real-time RT-PCR

Real-time quantitative RT-PCR was performed in an ABI 7500 sequence detection system (Applied Biosystems) using TaqMan Assays on Demand reagents for IL-17A, IL-17F, IL-21, IL-22, IL-23, Foxp3, GITR, TGFβ, CTLA-4, TLR1-10, Nod1, Nod2, NALP3 and an endogenous 18 s ribosomal RNA control. Relative transcripts were determined by the formula
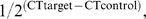
where CT is the threshold cycle during the exponential phase of amplification, according to the manufacturer's protocol.

### Statistical analysis

Comparisons were made using the Mann-Whitney U test. All statistics were performed using GraphPad Prism version 5 for Windows (GraphPad Software, Inc., San Diego, CA).

## Results

### Filarial antigen induces augmented Th1 responses in CP patients

**Figure 1:**
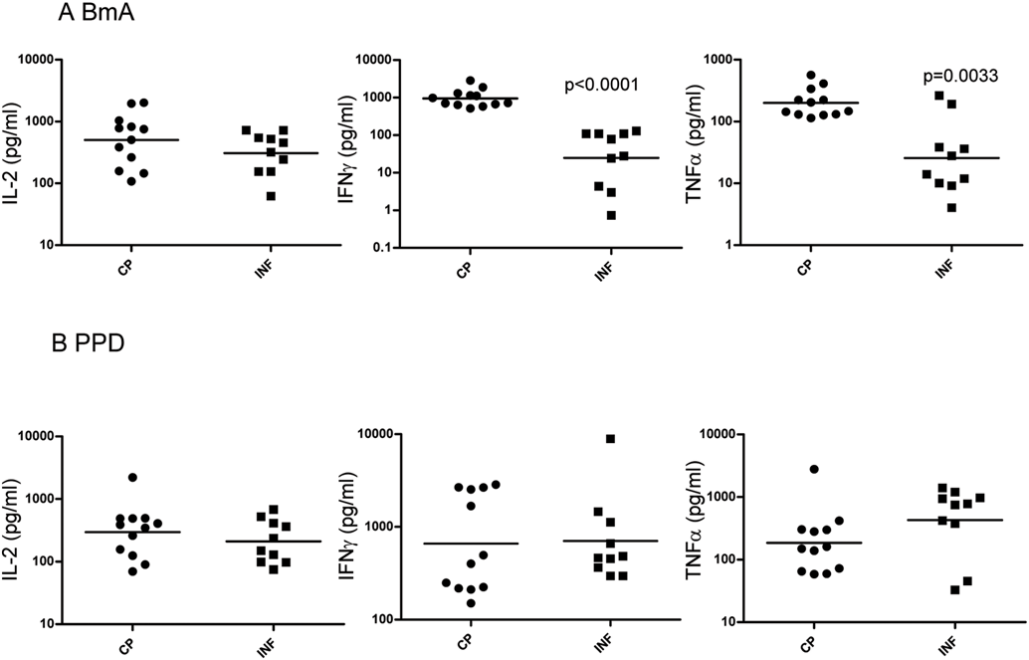
Filarial lymphedema is associated with elevated Th1 cytokine secretion. (A, B) PBMCs from filarial lymphedema [CP] (*n* = 12) and asymptomatic infected [INF] (n = 10) patients stimulated with (A) BmA (10 µg/ml) or (B) PPD (10 µg/ml) for 24 hours, and Th1 - IL-2, IFN-γ, and TNF-α cytokine levels were measured by ELISA. Results are shown as net cytokine production over media control. P values were calculated using the Mann-Whitney test.

To determine the role of Th1 cytokines in development of lymphedema, we stimulated PBMCs from CP and INF patients with BmA or PPD for 24 hours and measured the levels of prototypical Th1 cytokines by ELISA. As shown in Figure 1A, BmA induced significantly increased production of IFN-γ (geometric mean [GM] of 956.8 pg/ml in CP vs. 24.88 pg/ml in INF; P<0.0001) and TNF-α (GM of 200.2 vs. 25.64; P = 0.0033) but not IL-2 (GM of 501.5 vs. 308.5) in CP compared with INF. As shown in Figure 1B, no significant alteration in the production of IFN-γ (GM of 659.1 vs. 707.7), TNF-α (GM of 185.0 vs. 428.5), and IL-2 (GM of 296.5 vs. 210.8) was observed in response to the control antigen PPD. Thus, elevated Th1 responses in CP patients were filarial-antigen specific.

**Figure 2:**
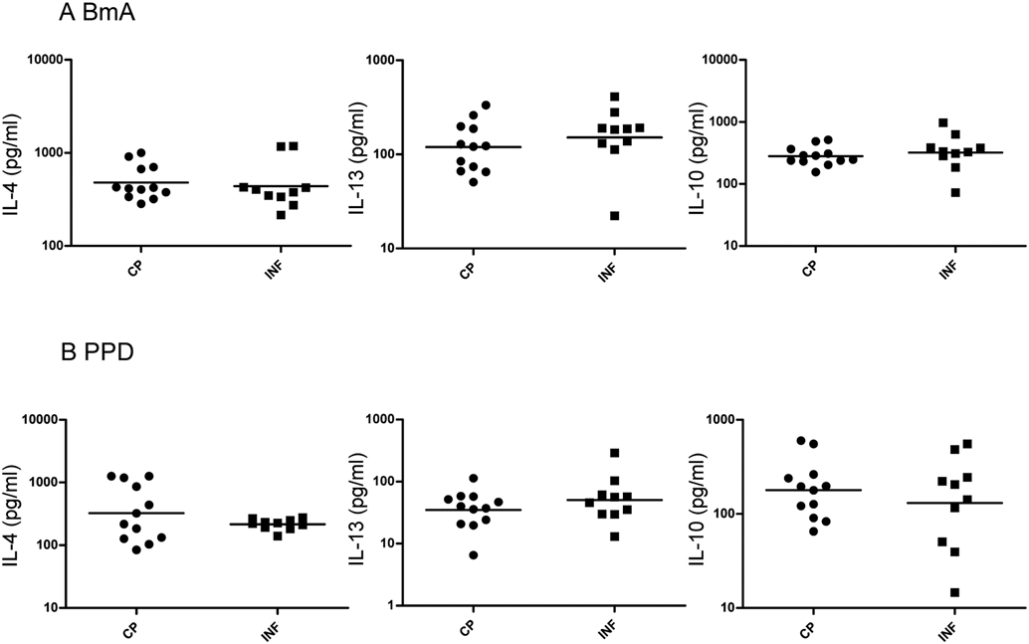
Filarial lymphedema is not associated with elevated Th2 cytokine secretion. (A, B) PBMCs from filarial lymphedema [CP] (n = 12) and asymptomatic infected [INF] (n = 10) patients stimulated with (A) BmA (10 µg/ml) or (B) PPD (10 µg/ml) for 24 hours, and Th2 - IL-4 and IL-13, as well as IL-10, cytokine levels were measured by ELISA. Results are shown as net cytokine production over media control. P values were calculated using the Mann-Whitney test.

### Filarial antigen does not induce differential Th2 or IL-10 responses in CP patients

Patients with lymphedema exhibited Th2 or IL-10 responses to filarial antigen that were no different than the Th2 or IL-10 responses seen in INF patients (Figure 2A). Upon stimulation with BmA, PBMCs from CP patients produced levels of IL-4 (GM of 480.0 vs. 438.2), IL-13 (GM of 119.2 vs. 151.7), and IL-10 (GM of 281.2 vs. 321.6) not significantly different from those from INF patients. Similarly, in response to PPD, no significant alteration in the levels of IL-4 (GM of 324.8 vs. 215.8), IL-13 (GM of 32.09 vs. 50.65), and IL-10 (GM of 178.9 vs. 130.4) was observed (Figure 2B).

### Filarial antigen induces enhanced expression of the IL-17 cytokine family in CP patients

**Figure 3:**
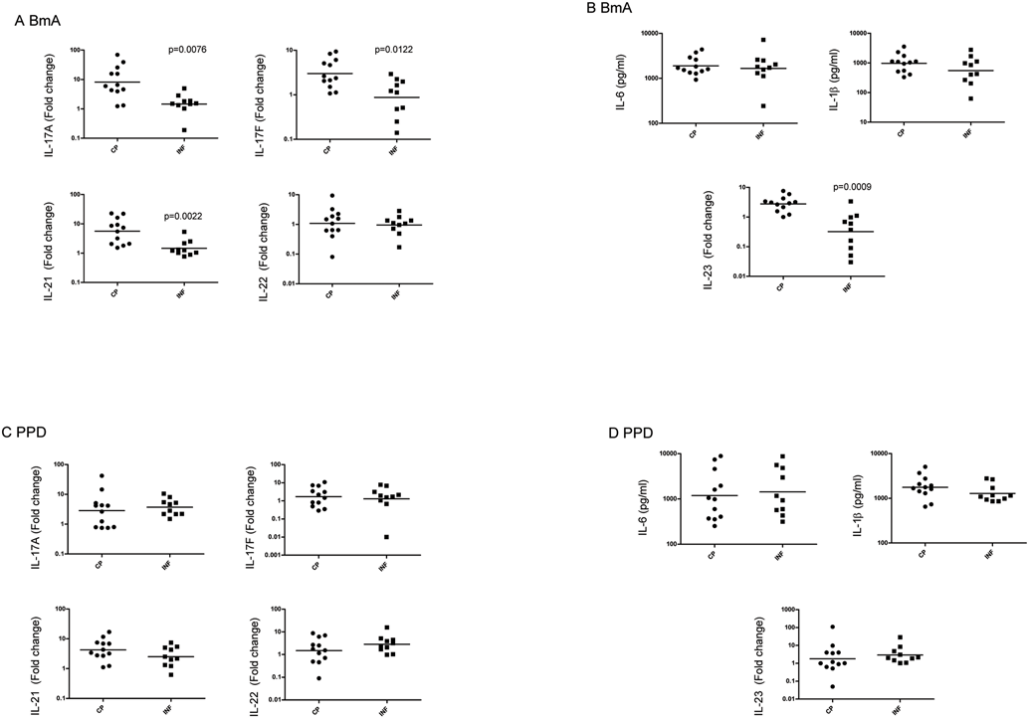
Filarial lymphedema is associated with enhanced expression of the IL-17 family of cytokines. PBMC expression of IL-17A, IL-17F, IL-21 and IL-22 mRNA as measured by real-time RT-PCR following 24-hour stimulation with (A) BmA or (C) PPD and PBMC secretion of IL-6 and IL-1β as measured by ELISA and IL-23 mRNA as measured by real-time RT-PCR following 24-hour stimulation with (B) BmA or (D) PPD in filarial lymphedema [CP] (n = 12) and asymptomatic infected [INF] (n = 10) patients. Results are depicted as net cytokine production or as fold change over media control. P values were calculated using the Mann-Whitney test.

To address the role of Th17 cells in filarial lymphedema, we examined the expression of IL-17A, IL-17F, IL-21 and IL-22 as well as the upstream inducing cytokines IL-23, IL-6 and IL-1β in PBMCs of lymphedema patients following BmA or PPD stimulation. As shown in Figure 3, PBMCs from CP patients exhibited significant upregulation of IL-17A (GM fold change of 8.125 vs. 1.456; P = 0.0076), IL-17F (GM fold change of 3.003 vs. 0.8710; P = 0.0122), IL-21 (GM fold change of 5.629 vs. 1.460; P = 0.0222), and IL-23 (GM fold change of 2.771 vs. 0.3222; P = 0.0009) mRNA expression compared with INF patients. Again, this upregulation was filarial-antigen specific, as there were no significant differences in the expression of IL-17A (GM fold change of 2.841 vs. 3.723), IL-17F (GM fold change of 1.711 vs. 1.293), IL-21 (GM fold change of 4.250 vs. 2.502), or IL-23 (GM fold change of 1.799 vs. 2.913) in response to PPD (Figure 3C and 3D) between the two groups. IL-22 was the only IL-17 family cytokine whose production did not differ between the two groups upon stimulation with BmA (GM fold change of 1.081 vs. 0.9531). In addition, BmA and PPD induced levels of cytokines upstream of the IL-17 axis - IL-6 (GM of 1895 pg/ml vs. 1664 pg/ml for BmA; 1189 vs. 1429 for PPD) and IL-1β (GM of 972.7 vs. 551.7 for BmA; 1758 vs. 1284 for PPD) that did not differ between the two groups.

### Impaired expression of nTreg markers in CP patients

**Figure 4:**
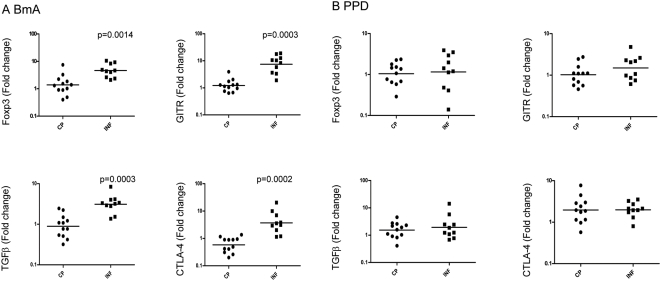
Filarial lymphedema is associated with lack of Foxp3, GITR, TGFβ, and CTLA-4 upregulation. (A, B) PBMCs from filarial lymphedema [CP] (n = 12) and asymptomatic infected [INF] (n = 10) patients were stimulated with (A) BmA (10 µg/ml) or (B) PPD (10 µg/ml) for 24 hours, and Foxp3, GITR, TGFβ, and CTLA-4 mRNA were measured by real-time RT-PCR. Results are shown as fold change over media control. P values were calculated using the Mann-Whitney test.

To determine the role of nTregs in the development of lymphedema, we stimulated PBMCs from CP and INF patients with BmA or PPD for 24 h and examined the mRNA expression of nTreg markers—Foxp3, GITR, TGFβ, and CTLA-4—by real-time RT-PCR. As shown in Figure 4A, CP exhibit significantly lower induction of Foxp3 (GM fold change of 1.377 vs. 4.1613; P = 0.0014), GITR (GM fold change of 1.223 vs. 7.462; P = 0.0003), TGFβ (GM fold change of 0.8928 vs. 3.118; P = 0.0003) and CTLA-4 (GM fold change of 0.5807 vs. 3.702; P = 0.0002) in response to BmA in comparison with INF. As shown in Figure 4B, no significant alteration in the expression of Foxp3 (GM fold change of 1.047 vs. 1.154), GITR (GM fold change of 1.022 vs. 1.488), TGFβ (GM fold change of 1.537 vs. 1.944) and CTLA-4 (GM fold change of 1.964 vs. 1.986) was observed in response to the control antigen, PPD. Thus, a failure of induction of nTregs was characteristic of antigen-driven responses in filarial lymphedema.

### Filarial antigen induces enhanced TLR expression in CP patients

**Figure 5:**
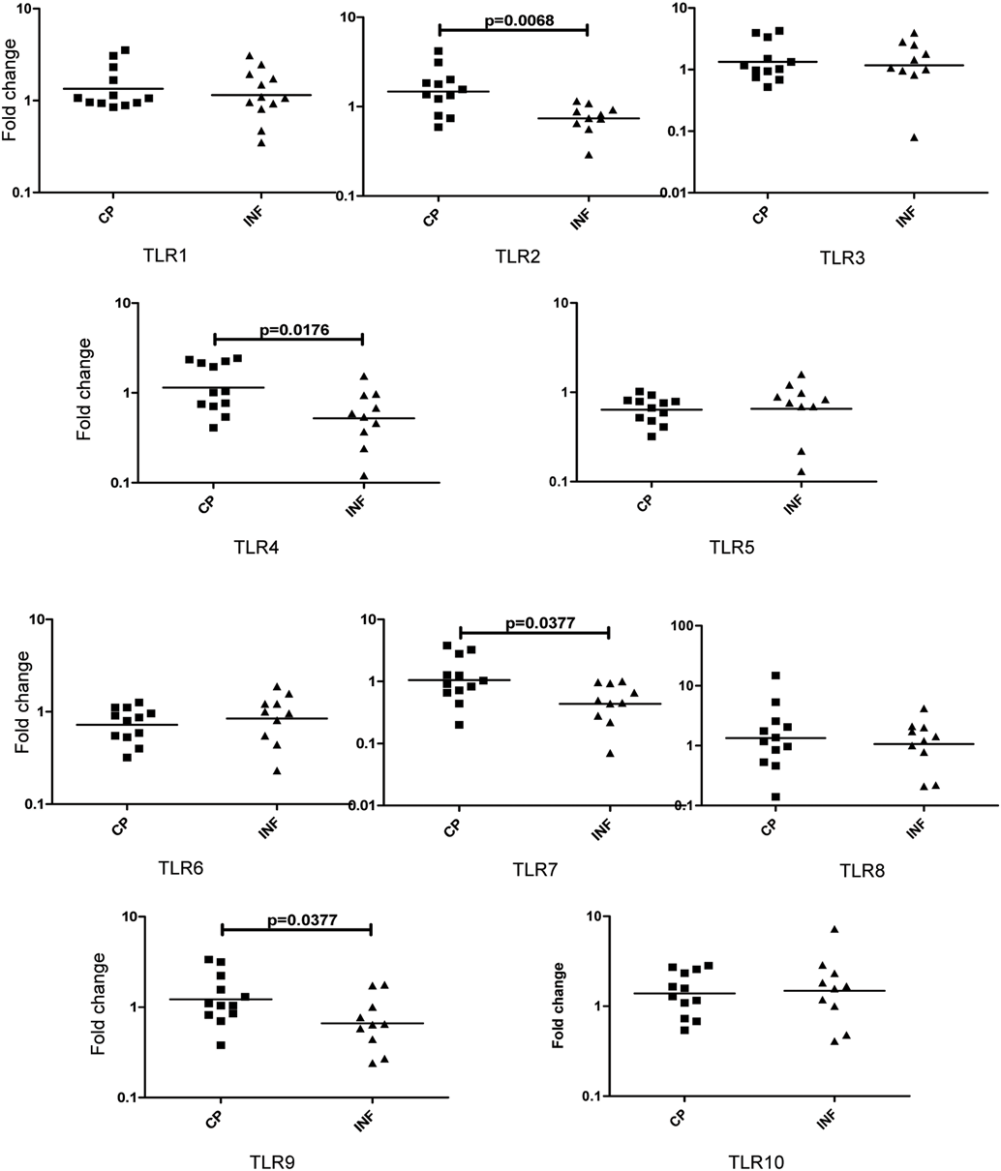
Filarial lymphedema is associated with increased expression of TLR2, 4, 7 and 9 mRNA. PBMCs from filarial lymphedema [CP] (n = 12) and asymptomatic infected [INF] (n = 10) patients were stimulated with BmA (10 µg/ml) for 24 hours, and TLR1-10 mRNA were measured by real-time RT-PCR. Results are shown as fold change over media control. P values were calculated using the Mann-Whitney test.

Because TLR have been implicated in development of pathology in animal models of filarial parasites as well as in a related filarial infection, onchocerciasis [4], we sought to determine the role of TLR expression in development of filarial lymphedema by examining the expression patterns of all ten human TLRs in PBMCs of CP and INF patients following 24-hour stimulation with BmA or PPD. As shown in Figure 5, CP exhibit significantly higher induction of BmA-stimulated TLR2 (GM fold change of 1.470 vs. 0.7357; P = 0.0068), TLR4 (GM fold change of 1.146 vs. 0.5216; P = 0.0176), TLR7 (GM fold change of 1.049 vs. 0.4361; P = 0.0377) and TLR9 (GM fold change of 1.217 vs. 0.6622; P = 0.0377) expression in comparison with INF. No significant alteration in the expression of other TLRs was observed in response to BmA (Figure 5). No significant difference in the expression levels of TLRs was observed in response to PPD stimulation (data not shown).

### Filarial antigen induces significantly increased expression of Nod1 and Nod2 in CP patients

**Figure 6:**
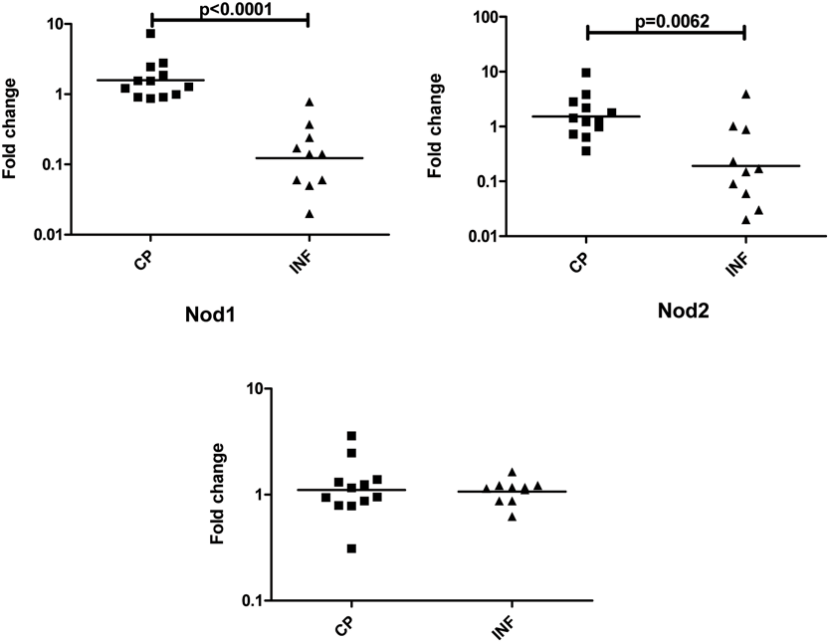
Filarial lymphedema is associated with increased expression of Nod1 and Nod2 mRNA. PBMCs from filarial lymphedema [CP] (n = 12) and asymptomatic infected [INF] (n = 10) patients were stimulated with BmA (10 µg/ml) for 24 hours, and Nod1, Nod2 and NALP3 mRNA were measured by real-time RT-PCR. Results are shown as fold change over media control. P values were calculated using the Mann-Whitney test.

NLRs are cytosolic proteins that play a role in regulation of proinflammatory pathways through NF-κB induced by bacterial ligands [17]. Three family members—Nod1, Nod2, and NALP3—have been shown to be particularly important in this process [17]. To elucidate the role of NLR in response to filarial antigen, we examined induction of Nod1, Nod2, and NALP3 following 24-hour stimulation with BmA or PPD in CP and INF patients (Figure 6). We found that BmA (but not PPD) induced significantly higher expression of Nod1 (GM fold change of 1.582 vs.0.1236; P<0.0001) and Nod2 (GM fold change of 1.518 vs. 0.1918; P = 0.0062) but not NALP3 (GM fold change of 1.108 vs. 1.071) in CP compared to INF patients.

## Discussion

The role of cytokines in inflammation is well established, and anticytokine therapies such as anti-TNF-α are now used routinely to treat chronic inflammatory diseases [18],[19]; however, to understand the underlying disease mechanisms so that more directed therapeutics might be developed, it was necessary to identify the cytokine networks that regulate the development of pathology. The Th1/Th2 framework has been useful in the understanding of infectious and allergic disease processes, but for inflammatory and autoimmune diseases, Th1 cells—clearly important in pathogenesis—are not the only subset promoting pathologic reactions [20]. Characterization of Th17 cells has furthered our understanding of the pathogenesis of many inflammatory and autoimmune disorders [20]. Th17 cells, characterized by expression of cytokines IL-17A and IL-17F as well as IL-21 and IL-22, are developmentally influenced in mice by IL-6, IL-21, IL-23, and TGFβ and in humans by IL-6, IL-1β, IL-23, and TGFβ [21]. Together with Th1 cells, Th17 cells play an important role in the establishment/maintenance of a variety of chronic inflammatory and autoimmune disorders [22]. Thus, chronic inflammatory responses in experimental autoimmune encephalomyelitis and collagen-induced arthritis are mediated by the IL-23-IL-17 axis [22], as are some of the immune-mediated pathology in murine schistosomiasis [23],[24] and toxoplasmosis [25]. nTregs are crucial in the maintenance of peripheral immune tolerance [26] and also play an important role in downregulating immune responses engendered by invading pathogens [27]. The ability to regulate potent immune responses following elimination of the pathogen is a major factor in limiting immune-mediated pathology in a variety of bacterial, viral, fungal and parasitic infections [27].

Lymphatic filariasis is a disease characterized by a broad spectrum of clinical manifestations including an asymptomatic (or subclinical) form seen among the majority of infected people [1]. The immune response in clinically asymptomatic individuals is dominated by IL-4, IL-5, IL-10, and IL-13 secretion, IgE and IgG4 antibodies, and peripheral eosinophilia [1]. A subset of individuals with this infection, however, has demonstrable pathology characterized, most notably, by lymphedema, hydrocele, and elephantiasis. To help elucidate whether filaria-induced pathogenic reactions were caused by unchecked pro-inflammatory (Th1/Th17) responses or by a failure to regulate these responses or pathology, we examined the contribution of Th1, Th2, Th17, and nTreg subsets in the development of filarial lymphedema.

Our finding that Th1 and Th17 responses are induced in patients with filarial pathology has clear implications. IFN-γ and TNF-α are potent stimulators of nitric oxide and other pro-inflammatory mediators of inflammation in a variety of cell types and hence could contribute directly to development of the pathology [28]. Th17 cells also contribute to inflammatory pathology through induction of IL-17A, IL-17F, IL-21, and IL-22 [22]. In our study, we found augmented induction of IL-17A, IL-17F, IL-21, and IL-23 in patients with filaria-induced lymphedema. IL-6 and IL-1β are two cytokines previously shown to be important for Th17 lineage commitment [20]; the fact that patients with or without pathology had equivalent production of these two cytokines suggests that establishment of the Th17 lineage is not what is altered in CP patients; however, the increased induction of IL-23 in these same patients does indicate that peripheral differentiation of committed Th17 cells is significantly enhanced. Most effector cytokines known to be produced by Th17 cells—IL-17A, IL-17F, and IL-21 (with the exception of IL-22)—are induced at significantly higher levels in patients with lymphedema, suggesting an important role for these cytokines in development of this disease process. Both Th1 and Th17 cells are known to induce production of two vascular endothelial growth factors of the VEGF family, VEGF-C and VEGF-D [29]. Enhanced production of VEGF-C has been implicated in development of filarial lymphedema [30],[31] and, hence, increased induction of Th1/Th17 responses might translate into increased VEGF-C production in the lymphedema patients in our study. Thus, the induction of these factors could have a direct effect on lymphatic vessel function or morphology leading to lymphatic dysfunction and secondary lymphedema. Study of expression of the VEGF family of molecules in filarial lymphedema patients is currently under way.

Pro-inflammatory pathology in a variety of settings is offset by the induction of a specific subset of T cells called natural or induced Tregs [26]. nTregs are characterized by expression of the transcription factor Foxp3, which is absolutely essential for the induction and maintenance of these cells [26]. GITR, a surface molecule present on nTregs, is known to contribute to the inhibitory function of these cells [32]. TGFβ and IL-10 are regulatory cytokines important in the induction of Treg activity as well [33]. Finally, CTLA-4 has been shown to be crucial in the maintenance of nTreg function [34]. We and others have previously shown that nTregs are important in the establishment of chronic infections in humans and animal models [35],[36]. Our results show that while Foxp3, GITR, TGFβ, and CTLA-4 are significantly upregulated in patients with asymptomatic infection, there is a significant lack of upregulation in filarial lymphedema patients. Hence, the pathogenesis of lymphatic pathology in filarial infections appears two pronged, with enhancement of pro-inflammatory networks (Th1 and Th17) on the one hand and depression of an anti-inflammatory subset of cells (nTreg) on the other.

When examining the pathways leading to initiation of the inflammatory cytokine cascade, the innate immune receptors commonly associated with initiation of inflammatory processes are the IL-1R- TLR and the NLR families [37],[38]. Studies in animal models of filarial infection and in vitro studies in humans have suggested that Wolbachia-derived molecules from filarial parasites are key inducers of proinflammatory cytokines [12],[13],[39]. Moreover, this inflammatory response to Wolbachia has been shown to be mediated primarily through TLR2, TLR4, and TLR6 [12],[40]. Indeed, TLR downregulation appears to be a mechanism of immune dysfunction in patent filarial infections [14],[15]. In addition, exposure of human dendritic cells to live filarial parasites has been shown to downregulate the expression and function of TLR3 and TLR4 [41]. The human TLR family includes ten members, TLR1–10, all of which play an important role in recognition of pathogen-associated molecular patterns. Our data reveal that modulation of TLR expression is an important feature of chronic lymphatic pathology since patients with lymphedema exhibit significantly enhanced expression of TLR2, 4, 7 and 9 mRNA in comparison to asymptomatic, infected patients. Increased expression of TLRs might potentially lead to increased expression of pro-inflammatory cytokines and promote pathology development. Studies to examine the functional effects of altered TLR gene expression in patients with filarial lymphedema are currently underway. A related family of pattern recognition receptors is the NLR, including Nod1, Nod2, and NALP3, among others. NLRs are intracellular proteins involved in recognition of intracellular pathogens as well as in activation of NF-κB and caspase-dependent pathways [42]. NLRs function as cytosolic sensors for induction of apoptosis, innate recognition of microorganisms, and regulation of inflammatory responses [42]. Our finding that Nod1 and Nod2 gene expression is upregulated in an antigen-specific manner in CP patients indicates that these cytosolic sensors are crucial in the inflammatory cascade associated with tissue damage in the lymphatics. Further experiments are needed to verify the cause-effect relationship between NLR family members and development of pathology in lymphatic filariasis.

In summary, our study examines in depth the complex cytokine patterns involved in the pathogenesis of filarial lymphedema. We have elucidated important roles for both Th1 and Th17 cells in driving the inflammatory pathology and have also unraveled a role for Tregs by demonstrating a lack of induction of these cells in patients with filarial lymphedema. Thus, increased induction of Th1 and Th17 cytokines with diminished activity of nTreg is clearly associated with development of lymphatic pathology. In addition, we have also discovered an association of pathology and cytokine production with transcriptional regulation of TLRs and NLRs, providing evidence that both membrane-bound and cytosolic pattern recognition receptors might be involved in inflammatory filarial pathology. Further elucidation of the exact roles played by these ‘sensors’ should provide new clues toward prevention of this debilitating disease through immune manipulation.
